# Flexible and Integrative Psychiatric Care Based on a Global Treatment Budget: Comparing the Implementation in Germany and Poland

**DOI:** 10.3389/fpsyt.2021.760276

**Published:** 2022-01-07

**Authors:** Julian Schwarz, Andrzej Cechnicki, Jan Godyń, Laura Galbusera, Daria Biechowska, Beata Galińska-Skok, Izabela Ciunczyk, Yuriy Ignatyev, Felix Muehlensiepen, Bettina Soltmann, Jürgen Timm, Sebastian von Peter, Marek Balicki, Jacek Wciórka, Martin Heinze

**Affiliations:** ^1^Department of Psychiatry and Psychotherapy, Brandenburg Medical School, Immanuel Clinic Rüdersdorf, Rüdersdorf, Germany; ^2^Community Psychiatry and Psychosis Research Centre, Jagiellonian University Medical College, Krakow, Poland; ^3^Psychosis Research Unit, Association for the Development of Community Psychiatry and Care, Krakow, Poland; ^4^Department of Public Health, Institute of Psychiatry and Neurology, Warsaw, Poland; ^5^Department of Psychiatry, Medical University of Bialystok, Białystok, Poland; ^6^Middle Pomeranian Mental Health Centre “Medison”, Koszalin, Poland; ^7^Centre for Health Services Research Brandenburg, Brandenburg Medical School, Rüdersdorf, Germany; ^8^Faculty of Health Sciences Brandenburg, Brandenburg Medical School, Neuruppin, Germany; ^9^Department of Psychiatry and Psychotherapy, Medical Faculty, Carl Gustav Carus University Hospital, Technical University Dresden, Dresden, Germany; ^10^Biometry Section, Competence Centre for Clinical Trials, University of Bremen, Bremen, Germany; ^11^Pilot Program Office of the National Mental Health Program, Institute of Psychiatry and Neurology, Warsaw, Poland

**Keywords:** integrated mental health care, home treatment, global budget payment, health service research, mental health systems research, process evaluation, complex intervention

## Abstract

**Background:** The past decade has witnessed the establishment of flexible and integrative treatment (FIT) models in 55 German and Polish psychiatric catchment areas. FIT is based on a global treatment budget (GTB), which integrates funding of all acute psychiatric hospital services for a regional population. Prior research has identified 11 specific program components of FIT in Germany. In this paper we aim at assessing the applicability of these components to the Polish context and at comparatively analysing FIT implementation in Poland and Germany.

**Methods:** Qualitative interviews about the applicability of the 11 FIT-specific components were conducted with the program managers of the Polish FIT models (*n* = 19). Semi-quantitative data on the FIT-specific components were then collected in 19 Polish and 10 German FIT models. We assessed the grading of each component, their overall degree of implementation and compared them between the two countries. In all study hospitals, structural and statistical parameters of service delivery were collected and compared.

**Results:** The qualitative results showed that the German FIT-specific components are in principle applicable to the polish context. This allowed the comparative assessment of components grading and degree of implementation, which showed only subtle discrepancies between German and Polish FIT models. The little discrepancies point to specific aspects of care such as home treatment, peer support, and cooperation with non-clinical and social welfare institutions that should be further integrated in the components' definition.

**Conclusions:** The specific program components of FIT as first defined from the German experience, serves as a powerful tool to measure, and evaluate implementation of integrated psychiatric care both within and between health systems.

## Introduction

In recent decades, health service providers in several countries have made extensive efforts to establish community crisis alternatives to inpatient psychiatric admission. In Europe and the UK there is now a broad spectrum of team-based, outreach and integrative care models for assisting people with severe mental illness (SMI) (1–4). In addition to acute day hospitals, residential crisis houses, and assertive community treatment (ACT), Crisis Resolution Teams (CRT) are probably the most widespread form of community-based acute treatment. However, only England and Norway have so far implemented CRT at the national level (5, 6). European Countries, such as the Netherlands or Switzerland, have introduced several forms of outreach care but do not yet offer it nationwide. Only England and Norway have so far implemented CRT at a national level (1, 7).

Germany and Poland are countries where acute psychiatric care is still predominantly provided in inpatient settings (8). There is evidence that daily- and performance-based remuneration, which is the predominant financing approach for German psychiatric inpatient care, leads to treating service users in rather costly inpatient settings (9). A similar state of affairs prevails in Poland, where only one quarter of mental health care expenditures are allocated for outreach care (10). In the past two decades, the psychiatric societies of both countries have endeavoured to establish framework conditions to enable the delivery of integrated psychiatric care. They have adopted a similar “Flexible and Integrative Treatment” (FIT) model, which has been implemented in hospitals of selected regions. The FIT model is based on a shift from a performance-based remuneration to a lump-sum global treatment budget (GTB). The GTB provides hospitals with the financial security and flexibility needed to develop more community-oriented, outpatient and outreach care, while at the same time reducing inpatient treatment days and enabling flexible shifts between different settings and intensities of care on a need-based basis (11, 12). Basic differences between standard and model care in the two countries are presented in Table 1. Because it significantly contributed to a reduction of coercive measures the German FIT Model is explicitly recommended by the recently published WHO “Guidance on Community mental health services” (13).

**Table 1:** Basic differences between standard psychiatric hospital care and flexible and integrative treatment (FIT) models in Germany and Poland.

**Country**	**Germany**		**Poland**	
**Psychiatric care model**	**FIT**	**Standard care**	**FIT**	**Standard care**
Adopting hospitals	22	385	33	172
Key unit of care model	Hospital based, setting-flexible teams, treating patients in up to four settings	Hospital based, separated teams for each treatment setting	Hospital based, setting-flexible teams, treating patients in minimum two settings	Hospital based, separated teams for each treatment setting
Mainly offered services and settings	Inpatient, day patient, outpatient, outreach care, acute outpatient care	Inpatient, daypatient, outpatient	Inpatient, day patient, outpatient, outreach care, acute outpatient care	Inpatient, daypatient, outpatient
Collaboration with social welfare institutions	Regular meetings between hospital and social welfare teams for integrated steering of services	not regular, not case based	Joint delivery of medical and social welfare services by CMHTs “Recovery plan”	separated approach
Payer	(Selected) statutory health insurance companies contracting for FIT	Statutory and private health insurance companies	National health insurance	National health insurance
Reimbursement of hospital services	Annual lump-sum, global budget for all patients treated	Fee for service, activity-based payment, disease related groups	Annual lump-sum, global budget per capita; for all inhabitants within catchment area	Fee for service, activity-based payment

*CMHT, Community Mental Health Team; FIT, Flexible and Integrated Treatment*.

### Legal Framework and Evolution of FIT in Germany

In Germany, integrated psychiatric care was first introduced with a legislative reform in 2000, allowing service providers—of both in- and outpatient sectors—to contract with selected statutory health insurance companies for a joint delivery of assertive outreach care (14). A GTB was first introduced in 2008 for one pilot region in rural northern Germany (15). It is negotiated between service providers and statutory health insurance companies and is established based on historical expenditures and on the number of patients to be treated. Thus, the GTB financing approach can be described as occupying a middle ground between block contracts (where providers are paid a fixed amount to deliver a specific, usually broadly-defined, service) and capitation (where providers receive lump-sum payments based on the number of patients treated) (16). After the positive evaluation of the pilot project, the model was applied to a legal framework (§64b social code V; FIT) that enables the development of one such model project in each German federal region. Importantly thus, FIT is not a concrete model of care but rather a legal framework, which can be flexibly adapted and implemented according to specific contexts, needs, and concepts of service providers. Instead of the usual performance-based remuneration, participating hospitals receive a GTB, with which they are obliged to offer a “continuous service provision across different settings, including a complex assertive outreach care.” By July 2021, 22 of these FIT models had been established, ensuring acute psychiatric care for 5.5 million people (8% of the adult German population).

### Legal Framework and Evolution of FIT in Poland

The development of FIT in Poland is based on a statutory health reform, namely the National Mental Health Program (NMHP), which was initiated in 2008. The key aim of the program was to reduce the need for inpatient wards by increasingly diverting to outpatient psychiatric care. This aim was pursued in two phases, namely first through the introduction of Community Mental Health Teams (CMHT; 2011–2015) and then of Mental Health Centres (MHCs; 2017–2021) in 33 selected catchment areas (17). MHCs form a steering unit that bundles and coordinates psychiatric and (in part) social services for people suffering from SMI in a particular region. A given MHC is sited at and administered by a psychiatric hospital department, whereas its services are mostly provided externally by CMHTs. Structural requirements and organisational standards of MHCs were recently specified and shall soon be established within the legal framework through an amendment of the NMHP. The Polish FIT models are funded through a GTB modality. From July 1, 2018, participating model regions receive a lump sum that is calculated according to the capitation principle, i.e., as the product of the supply costs per capita and the number of inhabitants in a region. Nowadays, 12% of the Polish adult population−3.8 million people—is covered by FIT models in 33 MHCs. The agreements for another 10 MHCs have already been signed, such that at the end of 2021 there shall be a total of 43 MHCs covering 15% of the adult Polish population. In the event of a positive evaluation, FIT model care is to be rolled out nationwide (2022–2027) (18).

### Previous Findings From FIT Models

The initial outcome evaluation studies of FIT (and of its precursor models) in Germany have shown a significant reduction of inpatient length of stay, as well as an increase of service users treated in the day-patient, outpatient and outreach settings (19–21). They have also shown improved personal continuity of care between settings and fewer instances of involuntary treatment or coercive measures (20, 22). The clinical outcomes (e.g., HoNOS, CGI, and GAF) of service users improved, whereas the overall costs for mental health care were kept stable or even decreased (19, 23, 24). Moreover, FIT models were positively evaluated by service users, caregivers, and clinical staff in Germany (25–28). Due to the more recent introduction of the Polish FIT models, results from observational studies are still pending. Results of a pilot outcome study indicate higher levels of satisfaction among patients using FIT compared to standard care (29).

The aim of this exploratory study is to examine comparatively the structures and processes of FIT models in Germany and Poland, and to address the following research questions:

1. What are the fundamental similarities and differences between service provision in the Polish and German models of integrated psychiatric care?
2. To what extent can specific program components of the German FIT models be adapted for assessing implementation in the Polish context?

## Methods

### Design

This study was carried out between February 2020 and July 2021 under the auspices of the Polish-German Society for Mental Health (PGSMH). The aim of this psychiatric society is to strengthen exchange in research, practise, and thus cooperation between the two countries (30).

As part of a previous process evaluation study (25), 11 empirically based, practicable, and quantifiable program components have been developed to describe treatment structures and processes of German FIT models (see Table 1) (31). Subsequently, these FIT-specific components were used routinely to measure, evaluate, and thus assure quality during the process of FIT model implementation in Germany (25, 26, 31–33). So far, there have been no comparable guidelines or instruments for the quality assurance of the newly introduced Polish FIT models. To address the first research question, we collected structural and performance data, which enabled a comprehensive comparison between the two countries and a contextualisation of the component grading. For the second question, we investigated the degrees of implementation of the 11 FIT-specific components in the two countries. The applicability of the components for the Polish context was examined beforehand using qualitative expert interviews. We did not seek or require ethical approval for this study, since only institutional and non-patient data was used.

### Setting and Sampling

We selected a total of 30 model regions each in Germany and Poland on the basis of defined structural criteria, which included population density in the catchment area and duration of the model project. Thereby we aimed at representing the broadest possible spectrum of different FIT implementations. Of the 22 clinics that had adopted FIT in Germany as of August 2021, 10 clinics participated in the study. These are located in Heide, Itzehoe, Glauchau, Lüneburg, Riedstadt, Berlin (districts of Kreuzberg and Neukölln), Rüdersdorf, Bonn and Bochum. The data from the German clinics (except from Rüdersdorf) had been collected as part of a nationwide study to evaluate the effectiveness, costs and processes of FIT models (“PsychCare” study, duration: 2017–2021) (34). Of the 33 clinics in Poland implementing FIT as of August 2021, 20 took part in the study. Only one centre was excluded from the analysis due to incomplete data delivery. The clinics are located in: Koszalin, Suwałki, Bolesławiec, Hajnówka, Sandomierz, Łomza, Bielsko-Biała, Nowa Deba, Toruń, Gorlice, Kraków, Warszawa (IPiN, Bródno, Wolski), Chełm, Radzyń Podlaski, Miedzyrzecz, Tarnów, Grajewo.

### Specific Program Components of FIT

Specific FIT components (see Table 2) enable us to assess the degree of implementation of FIT in a given hospital. The components were developed based on the German FIT models in a multi-stage process (operationalized, weighted, quantified, and validated) (31). Each component consists of one to four items, which are to be rated on a point scale of 0–2 points depending on their weight. From the single item values scores for components and a total score are calculated. The total score depicts the degree of implementation of FIT at the corresponding study centres. The assessment of components is conducted by administering a fully structured questionnaire, the questions of which are answered on the basis of performance and structural data from any FIT-adopting hospital. Further methodological details are provided by Johne et al. (31). A recent study confirms the fitness of the FIT-specific program components to differentiate statistically between FIT models and standard care (32).

**Table 2:** FIT-specific components and their operationalization according to Johne et al. (31).

**No**.	**Component**	**Operationalisation**	**Assessment**
I	Shifting in- to outpatient setting *Shift of treatment from I*^a^* towards D*^b^* and/or O*^c^**	•Number of outpatient CoT^d^/total number CoT^d^ during EP^e^	
II	Flexible care management across settings *Unproblematic shift of SoT*^f^* (prompt, little bureaucracy)*	•Number of CoT^d^ using all three SoT^f^ during EP^e^/ total number SoT^f^ •Treatment D^b^, I^a^, and/or O^c^ in the same unit (ward, level etc.) •Systematic steering of treatment beyond all SoT^f^ •Application of SoT^f^ spanning roster and therapy plans	Rating scale (0–2)
		•Number SoT^f^-spanning sessions (meetings etc.)	Rating scale (1–3)
III	Continuity of treatment team *Implementation of team- and individual-related continuity*	•Percentage of staff working in more than one SoT^f^ (on a regular basis) •Coordinated admission (coordinating staff member) •Coordination of treatment by e.g., case manager, SoT^f^-spanning care •Home treatment by I^a^- and D^b^- teams •Outsourced PIA (outpatient department) team (not working in I^a^ or D^b^)	Rating scale (0–2)
IV	Multiprofessional Cooperation *Intense multiprofessional cooperation*	•Absolute number of mandatory sessions across all occupational groups	Absolute number
		•Measure/action to optimise cooperation across all occupational groups	Rating scale (0–1)
		•Training sessions multiprofessional cooperation	
		•Number occupational groups working in home treatment (on a regular basis)	Rating scale (0–2)
V	Therapeutic group sessions across all settings *Therapeutic groups with members from all SoT*^f^**	•Number of group sessions open for all SoT^f^	Rating scale (0–2)
VI	Outreach home care *Multiprofessional treatment at home ≥ 1x week*	•Number CoT^d^ with home-treatment/ all I^a^-cases during EP^e^	Rating scale (0–2)
		•Cars for home-visits	
VII	Involvement of carers *Caregivers as therapeutic tool*	•“Network” or other forms of systemic dialog with caregivers and/or “carer-conference” and/or “caregiver groups”	Rating scale (0–1)
		•Number of groups open for carers	Rating scale (0–1)
		•Percentage of systemic training for staff/employees (e.g., open dialogue)	Percentage
VIII	Accessibility of services *Geographical accessibility and accessibility of teams*	•Accessibility of services within one-hour drive •24-h-accessibility of multiprofessional mental health team (not doctor on call or the like) •Shuttle service for services users	Rating scale (0–2)
		•Waiting list	Reverse rating scale (1–0)
IX	Sovereign steering of services *Freedom of therapeutic decisions*	•Number of exceeds ≥ 2 nights in a row during EP •Number of exceeds per service user/ calendar week during EP •D^b^ treatment as well during the night •Rules according to contract in all matters concerning setting of treatment and length of treatment	Rating scale (0–2)
X	Cooperation across sectors *Cooperation with ambulant care systems*	•Mutual scheduling and realising of treatment with ambulant care systems (Social Code V) •Mutual scheduling and realising of treatment with social welfare system (Social Code XII)	Rating scale (0–2)
		•“Community psychiatric network”	Rating scale (0–1)
XI	Expansion of professional expertise *Professionalisation of staff*	•Multiprofessional training of staff concerning FIT models •Measures to multiply knowledge about FIT models •FIT models as part of appraisal interviews	Rating scale (0–1)
		•Percentage of nurses/caregivers moderating group sessions	Percentage

a *I, inpatient*.

b *D, day-patient*.

c *O, outpatient*.

d *CoT, case of treatment*.

e *EP, evaluation period*.

f *SoT, setting of treatment (outpatient, day-patient, inpatient)*.

### Data Collection and Analysis

#### Qualitative Data

Before being able to grade the FIT-specific components in the Polish MHCs, we had to validate and confirm the applicability of the components in the Polish model regions. For this purpose, we initially carried out qualitative expert interviews with managers and program developers from all Polish study centres (35). The interviewers (JG, BG) had been trained by members of the German research team (JS, YI, SvP, MH) who had previously established the 11 specific program components of German FIT models. As a guideline for the qualitative interviews we used the questionnaire for the grading of the specific components, which had been translated into Polish language (JG, AC). Participants were asked to discuss the appropriateness of the (sub-)components for the Polish context and to denote any potential deviations and required additions. Qualitative data was then scrutinised using content analysis according to Mayring (35). Deviations of the Polish FIT models from the original operationalization of the FIT-specific components were thematically summarised (deductive approach).

#### Grading of FIT-Specific Components

Based on the qualitative findings the German and the Polish research teams agreed on one version of the FIT-specific components which was then applied in all study centres in Germany and Poland. For this purpose, we carried out structured telephone interviews with the management of each study centre, in which the grading of each component was assessed. To ensure a sufficiently uniform process in the two countries, all interviews were conducted by the same interviewers (JS for Germany, JG for Poland). Quantitative grading data were analysed in a series of steps: First, the components values and the total score were calculated for each centre and tabulated using descriptive statistics. The total score of overall FIT compliance was calculated as an equally weighted mean of each component value; in other words, all components were considered to be equally important dimensions of FIT implementation (31). We then tested whether the total scores differed significantly (*p* < 0.05%) between the German and the Polish study centres. This question was addressed deductively, with testing of the null hypothesis “the same total score values in both countries” using the Mann-Whitney test. Due to the exploratory nature of this study, we made no alpha adjustment. The analyses were carried out with the SYSTAT software, version 13. Component one had to be excluded from the evaluation, as insufficient parameters were available for its quantification (see Table 1). Missing values on the component grading (13 of a total of 1,008 values) were entered as “no positive information possible” after a data verification.

#### Structural and Performance Data

To be able to compare German and Polish study clinics and to contextualise FIT grading results, additional data were collected (e.g., population size of the catchment area, average length of stay) from each study region. Performance data parameters were calculated based on routine data at each centre and then transmitted to the study team. All parameters collected were grouped for Germany and Poland and if possible, mean and standard deviation, weighted by the number of cases or patients, were calculated.

## Results

### Structural and Performance Data

Structural and statistical parameters of the Polish and German FIT models are summarised in Table 3. The complete information on the individual centres can be found in the Supplementary Table 1.

**Table 3:** Structural and statistical parameters of service delivery in FIT models in Poland (“POL”) and Germany (“GER”).

	**POL**	**GER**
***Structural and environmental parameters***		
Model regions, present study, *N* (%)	19 (57.6)	10 (45.4)
Model regions, overall, *N* (%)	33 (100.0)	22 (100.0)
Runtime of model project (years; beginning - 30.09.2021); M (%)	3.1 (0.1)	7.1 (1.5)
Clinic type, *N*		
Department at a general hospital	11	6
Specialised hospital	8	4
University hospital	2	1
Sponsorship, *N*		
Public	19	8
Non-profit	1	2
Private	0	0
Share of the clinic budget that is negotiated as a model project		
90–100%	19	4
50–89%	0	0
30–49%	0	4
1−29%	0	2
Population density in catchment area (Inhabitants/km^2^)		
Rural (<200)	12	4
Suburban (200–2.000)	3	3
Urban (>2.000)	4	3
Average size of catchment area (in 1.000 inhabitants), M (SD)	100.4 (40.1)	324.6 (240.7)
Day clinic treatment places per 1.000 Inhabitants, M (SD)	0.233 (0.09)	0.239 (0.08)
Actual hospital beds per 1.000 inhabitants, M (SD)	0.312 (0.05)	0.466 (0.09)
***Service delivery parameter (data year: 2019)***		
Percentage of patients per setting, M^a^ (SD^a^)		
Inpatient	11.3 (6.8)	33.4 (16.3)
Day-patient	2.4 (0.9)	9.8 (5.1)
Outpatient	81.0 (14.3)	76.1 (17.6)
Outreach care	5.2 (4.4)	7.6 (13.9)
Percentage of patients who used, M^a^ (SD^a^)		
Two settings	1.84 (0.6)	6.2 (5.1)
Three settings	0.59 (0.5)	3.3 (3.4)
Cases per patient, M^a^ (SD^a^)		
Inpatient	1.15 (0.25)	1.42 (0.25)
Day-patient	1.52 (1.28)	1.25 (0.65)
Length of stay (days), M^a^ (SD^a^)		
Inpatient	23.3 (6.0)	22.1 (4.5)
Day-patient	48.7 (22.7)	32.2 (5.7)
Length of stay (days; cumulative per year), M^b^ (SD^b^)		
Inpatient	27.4 (6.3)	30.5 (7.7)
Day-patient	50.7 (16.1)	34.6 (7.6)

*M, Mean; SD, Standard deviation*.

a *Weighted by the total number of patients of each hospital*.

b *Weighted by the total number of cases of each hospital*.

In comparison to Germany, in Poland a relatively larger number of model regions has emerged in a much shorter period of time. In both countries, model clinics are mainly located in departments at publically owned general hospitals. The Polish centres have switched their entire care to model care, while the majority of German clinics provide model care only for patients covered by certain statutory health insurance companies. Polish models are mainly implemented in relatively less unpopulated catchment areas.

In both countries, the majority of patients receive outpatient treatment, although this proportion is slightly higher in Poland. On the other hand, the day-clinic setting is increasingly used in Germany. The proportion of patients being treated in two to three different settings is slightly higher in the German model regions.

### Qualitative Comparison of FIT-Specific Components

In summary, managers (*n* = 16) and program developers (*n* = 6) of the Polish models (*n* = 19) who participated in the survey rated all (sub-) components as generally suitable for the Polish context. However, some participants reported that certain FIT-specific components have a different relevance in Poland than in Germany and lack some aspects they deem crucial to the Polish FIT models. These differences are presented alongside the FIT-specific components as follows:

II. Flexible Care Management Across Settings: This component includes individual treatment plans that span different settings and are handed out weekly to service users. In the Polish FIT models, on the other hand, there is a recovery plan that extends beyond acute hospital treatment and contains therapy goals but does not include a daily planning of the therapy sessions.
III. Continuity of treatment team: The sub-component “Home treatment by inpatient and day-patient team” was deemed to be of secondary importance in the Polish models, as the outreach teams mostly work separately from clinical teams and provide a more community-based service.
IV. Multiprofessional Cooperation: Although this feature was recognised in the Polish models, it here concerns fewer specific measures in comparison to the ones provided in Germany (such as interdisciplinary team days).
V. Therapeutic group sessions across all settings: In the Polish models, therapeutic groups are usually not offered across different settings. Survey participants attributed this to a differing underlying therapeutic concept, whereby group processes may be disrupted by the simultaneous presence of acute (= inpatient) and less acute (e.g., outpatient) patients.
VI. Outreach home care: This component is operationalized in the German FIT-components as acute treatment with a minimum treatment intensity of one home visit per week. In Poland, on the other hand, the intensity is controlled flexibly (sometimes <1 contact per week), since the treatment tended to be community-based and non-acute.
VIII. Accessibility of services: The sub-component “waiting lists for patients with a request for admission” is mostly not implemented in the Polish FIT models, as the legal stipulation (NMHP) of Polish FIT explicitly calls for patients to receive care within 72 h after the first contact with mental health services.
X. Cooperation across Sectors: The division of the sub-components on cooperation between actors in the social and health systems applies to both countries. Yet, the term “sector” [sektor] is used in Polish only to delimit these two areas of care, while it is used vaguely in German (e.g., to differentiate between inpatient and outpatient care). Therefore, the Polish experts recommended using a more precise definition, such as “Cooperation across Institutions and Sectors.”
XI. Expansion of professional expertise: The training and employment of peer-support workers is a high priority in the Polish models but is not included in the current version of the FIT-specific components.

### Degree of Implementation of FIT Models

**Figure 1:**
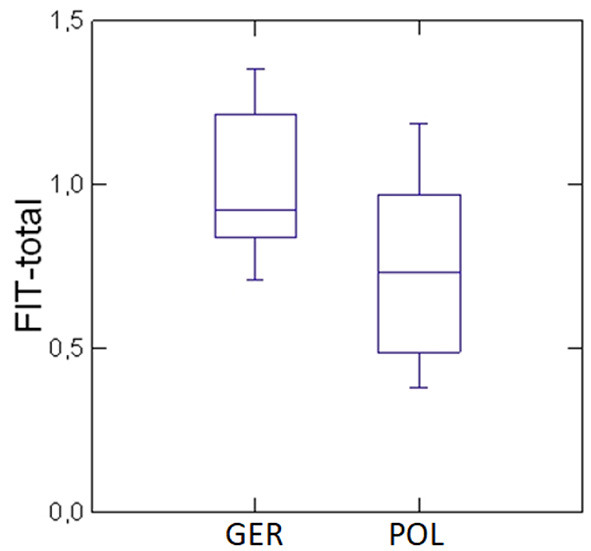
Comparison of the average overall degree of implementation (FIT total score) of the FIT-specific components in the German (“GER”) and Polish (“POL”) study centres. Box plot representation for the distribution of the overall degree of implementation: the bottom and top lines of the groups mark the minimum and maximum values, the horizontal marking within the two boxes represents the median and separates the box into a lower and an upper quartile.

With a mean total score of 0.99, the German clinics were slightly higher in the overall degree of implementation than the Polish clinics with 0.75 (see Figure 1). Although very small, this difference was statistically significant (*p* = 0.02). The individual total score of each study centre can be found in the Supplementary Figure 1.

**Figure 2:**
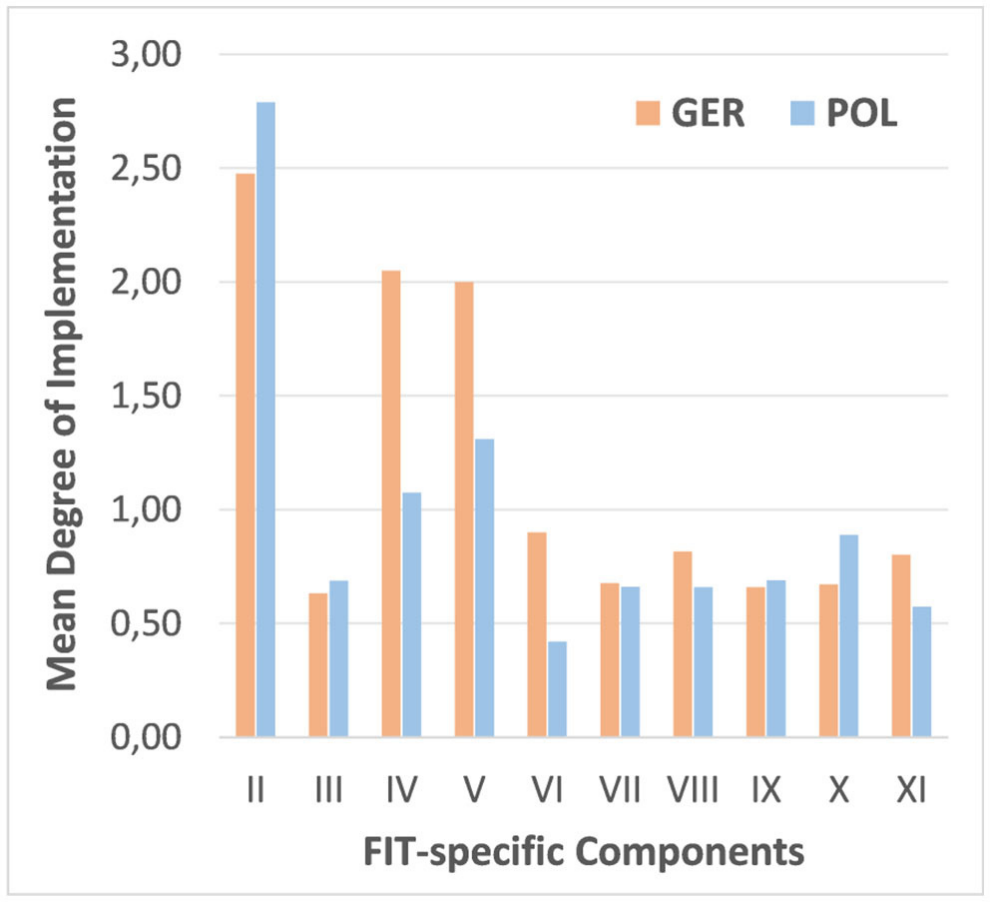
Average degree of implementation of the FIT-specific components in the German (“GER”) and Polish (“POL”) study centres.

The mean ratings of the individual components in Germany and Poland are presented in Table 4 and depicted in Figure 2. The components “Multi-professional Cooperation” (IV), “Therapeutic Group Sessions across all settings” (V) and “Outreach homecare” (VI) were rated significantly higher in the German models. These components may thus be particularly relevant for future FIT comparisons across Europe. Although the other differences were not statistically significant, results showed that German clinics scored higher in six of the FIT-specific components and the Polish clinics in four.

**Table 4:** Average rating of the FIT-specific program components in comparison between German and Polish model regions.

**Program component**	**Germany (*n* = 10)**	**Poland (*n* = 19)**	***p*-value**
	**Mean (SD)**		
II. Flexible care management across settings	2.48 (0.99)	2.79 (0.86)	0.5318
III. Continuity of treatment team	0.63 (0.33)	0.69 (0.33)	0.5809
IV. Multi-professional Cooperation	2.05 (0.96)	1.07 (0.32)	**0.0021**
V. Therapeutic group sessions across all settings	2.00 (0.00)	1.31 (0.69)	**0.0013**
VI. Outreach home care	0.90 (0.32)	0.42 (0.51)	**0.0202**
VII. Involvement of informal caregivers	0.68 (0.46)	0.66 (0.48)	0.9229
VIII. Accessibility of services	0.82 (0.20)	0.66 (0.27)	0.1013
IX. Sovereign steering of services	0.66 (0.24)	0.69 (0.35)	0.7412
X. Cooperation across Sectors	0.67 (0.38)	0.89 (0.54)	0.3039
XI. Expansion of professional expertise	0.80 (0.28)	0.57 (0.36)	0.1052

*SD, Standard deviation. Statistically significant values are shown in bold*.

## Discussion

The main result of the qualitative analysis is that the specific program components identified based on the German FIT models are generally suitable for describing the implementation of the newer Polish FIT models. We were able to operationalize and compare the characteristics of the FIT-specific program components in both countries, albeit with restrictions. Our comparison focuses on the differences between countries, and not between regions of a country.

A central aspect in the qualitative findings concerned the operationalization of outreach home care (part of components III and VI): The Polish models operate according to the principles of CMHT, i.e., with independent outreach teams that carry out home visits as required, but with adaptation of the frequency and duration of contacts based on service users' needs (17). On the other hand, outreach home care in the German FIT models is mainly grounded in a concept of CRT, which represents an alternative to inpatient admission. Consequently, home treatment is undertaken with a treatment intensity of more than one visit per week. This explains the significantly higher degree of implementation of this component by the German study centres.

In the German operationalization of component XI (Expansion of Professional Expertise) Peer Support Workers (PSW) are not included. In Poland, this professional group had existed at most of the sites since the advent of the FIT models (36). In the meantime, some of the German model clinics have also employed PSW (37). A case study in the German town of Geesthacht demonstrated the transformative power of the GTB financing approach for enabling the introduction of peer-supported interventions (38). PSW are therefore very important in FIT models of both countries. Future revisions of the FIT-specific components should allow a differentiated assessment of these aspects (including outreach treatment and peer support interventions) to better capture the current general states of development of the FIT models in each country.

The overall degree of implementation proved to be significantly higher in the German than in the Polish regions, presumably in relation to the two-fold longer mean run-time of the FIT models in Germany. This is in line with previous evaluation results showing a growing degree of implementation over the duration of a FIT model project, or of a similar care and remuneration model (26, 39). Nevertheless, the Polish models show a surprisingly high value for the overall and component-related degrees of implementation, given that they were introduced only in the past 3 years. We attribute this rapid implementation in Poland to a number of possible factors. Previous evaluations have shown that FIT models are particularly well-developed in German regions that offer a strong support in the implementation of FIT by health insurance providers and policy holders (26, 39). In Poland there has been a similar degree of acceptance and support of FIT models, which arguably contributed to the development of 33 models in just 3 years. Such support is also attested by the commitment of the Ministry of Health to start further model regions in the next few years.

German studies have indicated that clinics that have a FIT model contract with all health insurance companies implement the FIT components to a particularly high degree (25, 26, 39). This association also applies to all Polish models, since Poland has a single health insurance fund, i.e., the National Health Fund [NHF (Narodowy Fundusz Zdrowia)]. Therefore, if a FIT model is contracted in a Polish region, the responsible psychiatric clinic therefore reconfigures all their structures and processes from standard to model care. This enables the clinics to concentrate on service delivery according to the FIT model and ensures minimal friction or additional expenses that might otherwise occur due to the simultaneous operation of standard care (26).

From the perspective of health care systems, the present work reveals the advantages and disadvantages of a regulated market (RMS) vs. a national health system (NHS). Although by their formal typology, both countries qualify as RMS, the Polish NHF is more akin to an NHS. Since Poland has a single payer health service, it can presumably encourage reforms with greater effectiveness. In Germany, on the other hand, there is a patchwork of statutory health insurance companies. This makes it more difficult to implement system innovations—such as the FIT models—at the federal level, since all health insurance companies must first be convinced of its merits and have no obligation to enter such a contract. On the other hand, the possibility to conclude contracts with each single health insurance company—as it is in Germany—might enable a larger scope for small scale experimentation and for rapidly adopting new forms of care, since a decision need not occur at the federal level.

### Strength and Limitations

This is the first comparative study assessing FIT in two different countries. As a first limitation of this study, we note that only 10 of 22 (45%) of model regions in Germany participated in the survey. Nevertheless, the sample can be regarded as representative, because the study centres were selected to reflect the broadest possible spectrum of the model clinics (40). For the case of Poland, we obtained information across all regions.

The FIT-specific components were developed in 2016 for German model regions, but proved robust for use in Poland, requiring only minor linguistic changes. However, the models in both countries have evolved, such that certain features are missing (e.g., peer support) or are not operationalized with sufficient precision (e.g., home treatment). The qualitative survey made it possible to ensure that the existing components are generally suitable for use in the Polish model regions, with the acknowledgement of certain caveats.

Ten German experts contributed to weighting of the specific components for the original total scoring of the FIT model implementation (31). In the present analysis, we applied an equal weighting to each component for calculation of the total implementation score. For the further development of international FIT-specific components, a new weighting for each component should be jointly developed by the two countries.

Due to the naturalistic study design with only limited numbers of FIT-adopting hospitals in both countries, we cannot exclude the possibility of ß-errors when comparing results between Germany and Poland. In view of the identified qualitative differences between individual FIT components in the two countries, the composite value (FIT total score) could only approximately serve as a standard for comparison.

### Conclusions

The FIT-specific components were originally identified in Germany in 2016 in order to evaluate the introduction of innovative, cross-setting and ward-replacing treatment models (according to §64b social code V) and to promote quality assurance. A similar model of care has been piloted in Poland since 2018. With regard to the key research questions this study showed that 1. despite considerable health system differences between Germany and Poland, their psychiatric FIT models are very similar in terms of their core aspects of service provision; 2. the FIT-specific components are generally suitable for use in Poland, but they should be further supplemented and better specified to enable a more precise assessment of implementation differences between the two countries. In Poland and in Germany, decisions on the continuation or steadying of the FIT models in standard care will be pending in the next few years. Such decisions will require more scientific comparative knowledge about the evaluation and the implementation of FIT models also at the national level. The present findings constitute a first important step in this direction and point to the need for further research on integrative psychiatric care within the framework of Polish and German Mental Health Policy.

## Data Availability Statement

The datasets presented in this article are not readily available because of the used data protection declaration and the nature of qualitative interviews where individual participants could be possibly identified. Parts of the dataset are available from the research group on reasonable request. Requests to access the datasets should be directed to Julian Schwarz, julian.schwarz@mhb-fontane.de.

## Author Contributions

JS, MH, AC, and JT designed the study and applied for funding. JS, JG, and AC collected the qualitative and quantitative data and further structural and statistical data of service provision in the German and the Polish study regions. The qualitative analysis was conducted by JS. Statistical analyses were conducted by JT. JS drafted the first version of the manuscript, AC and MH supervised them. All authors are involved both in the preparation of the study and in its implementation, participated in the interpretation of the results, critically reviewed and commented on the manuscript, read and approved the final version of the manuscript.

## Funding

This work was funded by Brandenburg Medical School, Neuruppin, Germany.

## Conflict of Interest

The authors declare that the research was conducted in the absence of any commercial or financial relationships that could be construed as a potential conflict of interest.

## Publisher's Note

All claims expressed in this article are solely those of the authors and do not necessarily represent those of their affiliated organizations, or those of the publisher, the editors and the reviewers. Any product that may be evaluated in this article, or claim that may be made by its manufacturer, is not guaranteed or endorsed by the publisher.

## Abbreviations

ACT: Assertive Community Treatment
CMHT: Community Mental Health Teams
CRT: Crisis Resolution Team
FIT: Flexible and Integrated Treatment
GTB: Global Treatment Budget
MHC: Mental Health Centres
NHS: National Health System
NHF: National Health Fund
NMHP: National Mental Health Program
PSW: Peer Support Worker
RMS: Regulated Market System
SMI: Severe Mental Illness.

## References

[1] Stulz N Kawohl W Jäger M Mötteli S Schnyder U Hepp U. From research to practice: implementing an experimental home treatment model into routine mental health care. Eur Psychiatry. (2020) 63:e94. 10.1192/j.eurpsy.2020.91 33168129 PMC7681154

[2] Wheeler C Lloyd-Evans B Churchard A Fitzgerald C Fullarton K Mosse L. Implementation of the Crisis Resolution Team model in adult mental health settings: a systematic review. BMC Psychiatry. (2015) 15:74. 10.1186/s12888-015-0441-x 25879674 PMC4405828

[3] Odden S Landheim A Clausen H Stuen HK Heiervang KS Ruud T. Model fidelity and team members' experiences of assertive community treatment in Norway: a sequential mixed-methods study. Int J Ment Health Syst. (2019) 13:65. 10.1186/s13033-019-0321-8 31636700 PMC6796407

[4] Lloyd-Evans B Bond GR Ruud T Ivanecka A Gray R Osborn D. Development of a measure of model fidelity for mental health Crisis Resolution Teams. BMC Psychiatry. (2016) 16:427. 10.1186/s12888-016-1139-4 27905909 PMC5133753

[5] Hasselberg N Gråwe RW Johnson S Ruud T. An implementation study of the crisis resolution team model in Norway: are the crisis resolution teams fulfilling their role? BMC Health Serv Res. (2011) 11:96. 10.1186/1472-6963-11-96 21569226 PMC3116476

[6] Lloyd-Evans B Paterson B Onyett S Brown E Istead H Gray R. National implementation of a mental health service model: a survey of Crisis Resolution Teams in England. Int J Mental Health Nurs. (2018) 27:214–26. 10.1111/inm.12311 28075067

[7] Nugter MA Engelsbel F Bähler M Keet R van Veldhuizen R. Outcomes of FLEXIBLE assertive community treatment (FACT) implementation: a prospective real life study. Community Ment Health J. (2016) 52:898–907. 10.1007/s10597-015-9831-2 25648552 PMC5108818

[8] World Health Organization. Mental Health ATLAS 2017 Member State Profile. (2018). Available online at: https://www.who.int/mental_health/evidence/atlas/profiles-2017/en/ (accessed August 01, 2021).

[9] Schwarz J von Peter S Baumeister H Dahling V Gühne U Gouzoulis-Mayfrank E. DNVF-discussion paper - specificities, challenges and aims of mental health service research in Germany. Gesundheitswesen. (2021) 83:541–52. 10.1055/a-1478-3580 34169490

[10] National Health Fund headquarters. Prepared on the Basis of Data From the National Health Fund Headquarters as at 22.03.2021. (unpublished data) (2021).

[11] Schwarz J Schmid C Neumann A Pfennig A Soltmann B Heinze M. Implementing a global treatment budget for psychiatric hospital services - what are incentives, requirements, and challenges? Psychiatr Prax. (2021) 48:1–9. 10.1055/a-1421-3283 33902127

[12] Kroening-Roché J Hall JD Cameron DC Rowland R Cohen DJ. Integrating behavioral health under an ACO global budget: barriers and progress in Oregon. Am J Manag Care. (2017) 23:e303–9. 29087165

[13] WHO. Guidance on Community Mental Health Services: Promoting Person-Centred and Rights-Based Approaches. (2021). Available online at: https://www.who.int/publications/i/item/9789240025707 (accessed August 01, 2021).

[14] Amelung V Hildebrandt H Wolf S. Integrated care in Germany—a stony but necessary road! *Int J Integr Care*. (2012) 12:e16. 10.5334/ijic.853 22977429 PMC3429136

[15] Roick C Heinrich S Deister A Zeichner D Birker T Heider D. The regional psychiatry budget: costs and effects of a new multisector financing model for psychiatric care. Psychiatr Prax. (2008) 35:279–85. 10.1055/s-2008-1067432 18773374

[16] British Medical Association. Models for Paying Providers. (2018). Available online at: https://www.bma.org.uk/collective-voice/policy-and-research/nhs-structure-and-delivery/nhs-structures-and-integration/models-for-paying-providers (accessed August 01, 2021).

[17] Hat M Cechnicki A. Efficacy of home treatment of patients with mental disorders – a research review. Adv Psychiatry Neurol. (2021) 30:21–36. 10.5114/ppn.2021.106817 PMC9881613 37082031

[18] Pilot Program Office of the National Mental Health Program. Final Report on the Implementation of the Supervision and Control Work on the Implementation of the Pilot Programme of Community Psychiatry Under the National Programme for Mental Health Protection (unpublished typescript) (2021).

[19] Baum F Schoffer O Neumann A Seifert M Kliemt R March S. Effectiveness of global treatment budgets for patients with mental disorders—claims data based meta-analysis of 13 controlled studies from Germany. Front Psychiatry. (2020) 11:131. 10.3389/fpsyt.2020.00131 32265748 PMC7105704

[20] Assheuer M Beine K Mehl C Kellner M Agelink M Sieberer M. Umsetzung von Behandlungskontinuität im Versorgungsalltag – ein Vergleich zwischen zwei psychiatrischen Kliniken. Psychiatr Prax. (2020) 48:143–8. 10.1055/a-1274-3792 33232978

[21] Budnick A Kuhnert R Schmidt H Wienprecht L Kuhlmey A Blüher S. Sekundärdatenanalyse initial vollstationär behandelter Patienten mit Schizophrenie in einem Berliner Modellprojekt (nach § 64b SGB V). Gesundheitswesen. (2021) 83:936–45. 10.1055/a-1305-9991 33461237

[22] Wullschleger A Berg J Bermpohl F Montag C. Can “model projects of need-adapted care” reduce involuntary hospital treatment and the use of coercive measures? Front Psychiatry. (2018) 9:168. 10.3389/fpsyt.2018.00168 29765339 PMC5939233

[23] Berghöfer A Hubmann S Birker T Hejnal T Fischer F. Evaluation of quality indicators of integrated care in a regional psychiatry budget - a pre-post comparison by secondary data analysis. Int J Integr Care. (2016) 16:17. 10.5334/ijic.2479 28413369 PMC5354218

[24] König H-H Heider D Rechlin T Hoffmann P Birker T Heinrich S. How does the regional psychiatry budget (RPB) work in an area with initially low capacity of psychiatric hospital beds? Psychiat Prax. (2013) 40:430–8. 10.1055/s-0033-1343186 23695948

[25] von Peter S Ignatyev Y Johne J Indefrey S Kankaya OA Rehr B. Evaluation of flexible and integrative psychiatric treatment models in Germany—a mixed-method patient and staff-oriented exploratory study. Front Psychiatry. (2019) 9:785. 10.3389/fpsyt.2018.00785 30723433 PMC6349706

[26] Schwarz J Galbusera L Bechdolf A Birker T Deister A Duve A. Changes in German mental health care by implementing a global treatment budget—a mixed-method process evaluation study. Front Psychiatry. (2020) 11:426. 10.3389/fpsyt.2020.00426 32523551 PMC7261866

[27] Ignatyev Y Timm J Heinze M Indefrey S von Peter S. Development and preliminary validation of the scale for evaluation of psychiatric integrative and continuous care - patient's version. Front Psychiatry. (2017) 8:162. 10.3389/fpsyt.2017.00162 28912735 PMC5583144

[28] Schwarz J Duve A Hoffmann S Heiser P Heinze M von Peter S. Stakeholders‘ experiences with flexible and integrative treatment models in German child and adolescent psychiatry according to § 64b SGB V – a qualitative study. Zeitschrift für Kinder und Jugendpsychiatrie und Psychotherapie. (2020) 48:358–68. 10.1024/1422-4917/a000716 32122246

[29] Hat M Arciszewska-Leszczuk A Cechnicki A. Satisfaction with care in patients with schizophrenia treated in a pilot-program model and traditional care. Psychiatria Polska. (2021) 248:1–16. 10.12740/PP/OnlineFirst/138995 37350714

[30] Cichocki Ł Cechnicki A. Contribution of the Polish-German Mental Health Society to changes in Polish psychiatry. Psychiatr Pol. (2014) 48:395–400. 25016775

[31] Johne J von Peter S Schwarz J Timm J Heinze M Ignatyev Y. Evaluation of new flexible and integrative psychiatric treatment models in Germany- assessment and preliminary validation of specific program components. BMC Psychiatry. (2018) 18:278. 10.1186/s12888-018-1861-1 30176836 PMC6122621

[32] Schwarz J Ignatyev Y Baum F Neumann A Soltmann B Pfennig A. [Flexible and integrative treatment in psychiatry: implementation of specific care components at model and standard care clinics in Germany (PsychCare study)]. Nervenarzt. (2021). 10.1007/s00115-021-01238-2. [Epub ahead of print]. 34874468 PMC9061660

[33] von Peter S Ignatyev Y Indefrey S Johne J Schwarz J Timm J. Specific components for integrative and flexible care models according to § 64b SGB V. Nervenarzt. (2017) 89:559–64. 10.1007/s00115-017-0459-z 29209751

[34] Soltmann B Neumann A March S Häckl D Kliemt R Baum F. Multiperspective and multimethod evaluation of flexible and integrative psychiatric care models in Germany: study protocol of a prospective controlled multicenter observational study (PsychCare). Front Psychiatry. (2021) 12:659773. 10.3389/fpsyt.2021.659773 34140902 PMC8205541

[35] Mayring P. Qualitative Content Analysis: Theoretical Foundation, Basic Procedures Software Solution. Klagenfurt: SSOAR Open Access Repository (2014). 143 p. Available online at: http://nbn-resolving.de/urn:nbn:de:0168-ssoar-395173 (accessed August 01, 2021).

[36] Cechnicki A Liberadzka A. New role of people suffering from mental illnesses in treatment and recovery. Psychiatr Pol. (2012) 46:995–1005. 23479941

[37] Schwarz J Zeipert M Ignatyev Y Indefrey S Rehr B Timm J. Implementation and Stakeholders' Experiences With Home Treatment in Germany's Integrative and Flexible Psychiatric Care Models - A Mixed-Methods Study. Psychotherapie Psychosomatik Medizinische Psychologie. (2020) 70:65–71. 10.1055/a-0942-2163 31315143

[38] von Peter S Göppert L Ziegenhagen J Beeker T Glück R Groth B. Supported employment, participation at work, and peer support: a qualitative, participatory case study report of the Geesthacht model. Front Psychiatry. (2021) 12:634080. 10.3389/fpsyt.2021.634080 33967854 PMC8102772

[39] von Peter S Schwarz J Bechdolf A Birker T Deister A Ignatyev Y. Implementation of New Flexible and Integrative Psychiatric Care Models (According to §64b SGB V) in Rural Northern Germany in Comparison to Federal Territory. Gesundheitswesen. (2021) 83:33–9. 10.1055/a-0945-9851 31311061

[40] Petzold T Neumann A Seifert M Küster D Pfennig A Weiß J. Identification of control hospitals for the implementation of the nationwide and standardized evaluation of model projects according to § 64b SGB V: analysis of data from structured quality reports. Gesundheitswesen. (2019) 81:63–71. 10.1055/s-0042-116436 27846670

