# Supplementary material for: Flexible and Integrative Psychiatric Care Based on a Global Treatment Budget: Comparing the Implementation in Germany and Poland
Source: Front Psychiatry. 2022 Jan 7;12:760276. doi: 10.3389/fpsyt.2021.760276 (PMC8777040; doi:10.3389/fpsyt.2021.760276)

**Supplementary Tables and Figures**

**Supplementary Table S1**. Structural and statistical Data of participating study centres in Germany and Poland (data year: 2019)

| Structural and environmental parameters | German FIT Models | | | | | | | | | |
| --- | --- | --- | --- | --- | --- | --- | --- | --- | --- | --- |
| Hospital ID | 1 | 2 | 3 | 4 | 5 | 6 | 7 | 8 | 9 | 10 |
| Start of Model project (M/YY) | 1/14 | 1/16 | 10/16 | 1/15 | 1/13 | 1/14 | 1/13 | 1/16 | 1/16 | 1/13 |
| Clinic type (D = Department at a general hospital; H = Specialized hospital; U = University hospital) | D | H | H | H, U | D | H | D | D | D | D |
| Sponsorship (P = Public, N= Non-Profit) | N | P | P | P | P | N | P | P | P | P |
| Share of the clinic budget that is negotiated as a model project | 30 % | 100 % | 45 % | 40,67 % | 100 % | 33 % | 100 % | 10 % | 8,5 % | 100 % |
| Population density in catchment area (Inhabitants/km²) | 95 | 525 | 719 | 2.510 | 124 | 137 | 342 | 13.819 | 7.301 | 93 |
| Size of Catchment Area (in 1.000 inhabitants) | 235 | 330 | 950 | 260 | 135 | 436 | 130 | 289 | 330 | 150 |
| Day clinic treatment places per 1.000 Inhabitants | 0,29 | 0,16 | 0,11 | 0,17 | 0,36 | 0,24 | 0,31 | 0,19 | 0,28 | 0,29 |
| Hospital beds per 1.000 Inhabitants | 0,35 | 0,39 | 0,56 | 0,55 | 0,44 | 0,46 | 0,54 | 0,57 | 0,47 | 0,32 |
| ***Service delivery parameter (data year: 2019)*** | | | | | | | | | | |
| Percentage of Patients per setting  Inpatient  day-patient  Outpatient  Outreach care | 20,8  17,4  83,7  1,6 | 22,9  6,6  88,4  x | 38,6  8,4  80,4  x | 67,7  26,5  x  x | 75,4  19,1  62,1  2,4 | 24,1  10,7  84,8  2,9 | 35,2  3,8  39,2  x | 94,4  5,6  x  81,3 | 93,2  6,8  x  x | 31,7  15,2  41,5  11,5 |
| Percentage of Patients who used  two settings  three settings | 12,6 2,3 | 3,2 2,2 | 6,3 2,1 | x x | 9,1 5,0 | 9,3 4,3 | 3,6 4,9 | 1,9 1,4 | 1,8 1,3 | x x |
| Cases per Patient  Inpatient  day-patient | 1,46 1,24 | 1,26 1,10 | 1,42 1,02 | 1,72 1,07 | 1,05 x | 1,68 1,22 | 2,02 x | 1,28 1,14 | 1,57 1,12 | 1,45 1,23 |
| Length of stay (days)  Inpatient  day-patient | 21,4 22,8 | 26,5 39,0 | 26,6 31,2 | 20,5 27,8 | 15,0 x | 21,7 35,1 | 17,7 25,9 | 19,0 37,2 | 18,3 36,9 | 15,9 x |
| Length of stay (days; cumulative per year)  Inpatient  day-patient | 31,2 28,2 | 33,5 42,8 | 37,9 32,2 | 20,2 26,4 | 16,2 23,2 | 36,5 42,9 | 24,2 29,7 | 19,2 38,1 | 19,1 41,0 | 16,1 x |

x = Data not provided

| Structural and environmental parameters | Polish FIT Models | | | | | | | | | | | | | | | | | | |
| --- | --- | --- | --- | --- | --- | --- | --- | --- | --- | --- | --- | --- | --- | --- | --- | --- | --- | --- | --- |
| Hospital ID | 11 | 12 | 13 | 14 | 15 | 16 | 17 | 18 | 19 | 20 | 21 | 22 | 23 | 24 | 25 | 26 | 27 | 28 | 29 |
| Start of Model project | 9/18 | 7/18 | 7/18 | 7./18 | 8/18 | 7/18 | 9/18 | 10/18 | 10/18 | 10/18 | 10/18 | 10/18 | 11/18 | 10/18 | 10/18 | 9/18 | 12/18 | 10/18 | 7/18 |
| Clinic type  (D = Department at a general hospital; H = Specialized hospital; U = University hospital) | H | H | H | D | H | D | H | D | D | H | D, U | H | D | D, U | D | D | H | D | D |
| Sponsorship  (P = Public, N= Non-Profit) | P | P | P | P | P | P, N | P | P | P | P | P | P | P | P | P | P | P | P | P |
| Share of the clinic budget that is negotiated as a model project (%) | 100 | 100 | 100 | 100 | 100 | 100 | 100 | 100 | 100 | 100 | 100 | 100 | 100 | 100 | 100 | 100 | 100 | 100 | 100 |
| Population density in catchment area (Inhabitants/km²) | 87 | 39 | 56 | 27 | 95 | 67 | 1.125 | 98 | 1.440 | 91 | 5.462 | 5.141 | 60 | 4.253 | 6.142 | 44 | 30 | 1.269 | 40 |
| Size of Catchment Area (in 1.000 inhabitants) | 90,48 | 84,39 | 74,20 | 121,02 | 65,22 | 92,22 | 140,98 | 84,00 | 168,05 | 87,00 | 118,75 | 184,00 | 117,25 | 103,20 | 134,34 | 48,36 | 47,90 | 93,00 | 38,94 |
| Day clinic treatment places per 1.000 Inhabitants | 0,14 | 0,18 | 0,34 | 0,07 | 0,31 | 0,22 | 0,20 | 0,24 | 0,21 | 0,17 | 0,41 | 0,11 | 0,30 | 0,34 | 0,31 | 0,17 | 0,25 | 0,16 | 0,31 |
| Hospital beds per 1.000 Inhabitants | 0,28 | 0,31 | 0,34 | 0,29 | 0,32 | 0,4 | 0,39 | 0,32 | 0,31 | 0,33 | 0,24 | 0,35 | 0,32 | 0,2 | 0,3 | 0,3 | 0,35 | 0,29 | 0,3 |
| ***Service delivery parameter (data year: 2019)*** | | | | | | | | | | | | | | | | | | | |
| Percentage of Patients per setting  Inpatient  day-patient  Outpatient  Outreach care | 10,0  2,7  75,6  11,7 | x  x  x  x | 24,1  4,0  76,0  8,5 | 7,2  0,9  88,7  1,6 | 20,7  2,2  58,9  2,0 | 7,6  1,7  86,2  2,2 | 6,9  2,3  91,8  1,3 | 11,4  2,0  93,8  1,2 | x  x  x  x | 9,7  1,1  41,3  5,3 | 5,7  2,3  91,6  6,1 | 11,9  2,3  89,1  4,2 | 31,5  2,8  72,1  7,4 | 8,7  5,1  85,7  7,0 | 6,6  2,9  85,1  5,4 | 12,6  1,8  109,5  4,6 | 9,2  2,1  76,7  20,4 | x  x  x  x | 14,7  2,2  79,8  3,4 |
| Percentage of Patients who used  two settings  three settings | x  x | x  x | x  x | 0,9  0,8 | x  x | 1,9  0,3 | 1,6  0,5 | 1,8  0,5 | x  x | x  x | 1,7  0,3 | x  x | 2,5  2,0 | 2,3  0,1 | 2,5  0,6 | 1,4  0,4 | 0,7  0,1 | x  x | 3,4  0,0 |
| Cases per Patient  Inpatient  day-patient | 1,24  1,05 | x  x | 1,40  1,90 | 1,21  1,00 | 1,27  1,24 | x  x | 1,16  1,24 | 1,22  1,56 | x  x | 0,29  0,18 | 1,06  6,89 | 1,33  1,17 | 1,07  1,35 | 1,17  1,09 | x  x | 1,204  2,80 | 1,23  1,35 | x  x | 1,36  1,41 |
| Length of stay (days)  Inpatient  day-patient | 26  36 | x  x | 13  43 | 26  90 | 25  36 | 26,9  47 | 24,9  171 | 23  32 | x  x | 18,9  67,9 | 45  10 | 28  55 | 22,3  61,4 | 39  74 | 17,5  45,2 | 32  35 | 49  60 | x  x | x  x |
| Length of stay (days; cumulative per year)  Inpatient  day-patient | x  x | x  x | 36  83 | x  x | 25  36 | x  x | 26,8  x | 28  57 | x  x | 32  x | 48  68 | 38  64 | 24  65 | x  x | 21,9  x | 38  98 | x  x | x  x | 18,4  33,9 |

**Supplementary Figure S1.** Overall degree of implementation (FIT total score) of the FIT-specific components in the German (“GER”) and Polish (“POL”) study centres


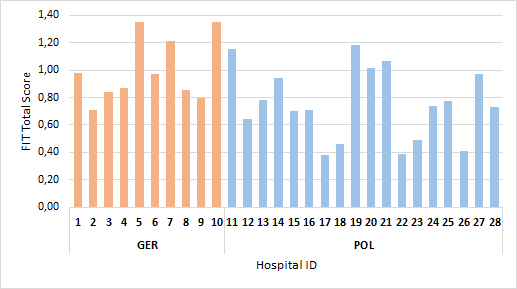

Supplement: Supplementary file 1 [file Data_Sheet_1.docx]
